# Preoperative respiratory muscle training combined with aerobic exercise improves respiratory vital capacity and daily life activity following surgical treatment for myasthenia gravis

**DOI:** 10.1186/s13019-023-02283-5

**Published:** 2023-04-24

**Authors:** Sai Chen, Xin Li, Yunshan Wu, Yana Li, Peili Cao, Yuchun Yin, Zhenguang Chen

**Affiliations:** 1 Center for Private Medical Service and Healthcare, The First Affiliated Hospital of Sun Yat-Sen University, Guangzhou, 510080 Guangdong People’s Republic of China; 2 Department of Rehabilitation, The First Affiliated Hospital, Sun Yat-Sen University, Guangzhou, 510080 Guangdong People’s Republic of China; 3 Department of Cardiothoracic Surgery of East Division, The First Affiliated Hospital, Sun Yat-Sen University, Guangzhou, 510700 Guangdong People’s Republic of China; 4 Present Address: Department of Thoracic Surgery, The First Affiliated Hospital, Sun Yat-Sen University, No. 58, Zhongshan Road II, Guangzhou, 510080 Guangdong People’s Republic of China

**Keywords:** Myasthenia gravis, Thymectomy, Aerobic exercise, Vital capacity, Exercise tolerance

## Abstract

**Objective:**

The effects of preoperative respiratory muscle training (RMT) on postoperative complications in patients surgically treated for myasthenia gravis (MG) remain unclear. The present study therefore evaluated the effects of preoperative moderate-to-intense RMT and aerobic exercise, when added to respiratory physiotherapy, on respiratory vital capacity, exercise capacity, and duration of hospital stay in patients with MG.

**Methods:**

Eighty patients with MG scheduled for extended thymectomy were randomly divided into two groups. The 40 subjects in the study group (SG) received preoperative moderate-to-intense RMT and aerobic exercise in addition to respiratory physiotherapy, whereas the 40 subjects in the control group (CG) received only chest physiotherapy. Respiratory vital capacity (as determined by VC, FVC, FEV1, FEV1/FVC, and PEF) and exercise capacity (as determined by the 6-min walk test [6 MWT]) were measured pre- and postoperatively and before discharge. The duration of hospital stay and activity of daily living (ADL) were also determined.

**Results:**

Demographic and surgical characteristics, along with preoperative vital capacity and exercise capacity, were similar in the two groups. In the CG, VC (*p* = 0.001), FVC (*p* = 0.001), FEV1 (*p* = 0.002), PEF (*p* = 0.004), and 6MWT (*p* = 0.041) were significantly lower postoperatively than preoperatively, whereas the FEV1/FVC ratio did not differ significantly. Postoperative VC (*p* = 0.012), FVC (*p* = 0.030), FEV1 (*p* = 0.014), and PEF (*p* = 0.035) were significantly higher in the SG than in the CG, although 6MWT results did not differ. ADL on postoperative day 5 was significantly higher in the SG than in the CG (*p* = 0.001).

**Conclusion:**

RMT and aerobic exercise can have positive effects on postoperative respiratory vital capacity and daily life activity, and would enhance recovery after surgery in MG patients.

## Introduction

Myasthenia gravis (MG) is a specific autoimmune disease affecting the muscles. MG is characterized by varying degrees of weakness in the respiratory, limb skeletal, swallowing, vocal and eyelid muscles. Its pathogenesis is closely related to pathological changes in thymus tissue, including thymic hyperplasia or thymic tumor, with 60–75% of MG patients having thymic hyperplasia and 20–25% having thymomas [1–3]. At present, surgery is the important treatment for MG [3, 4]. Although minimally invasive methods of thymectomy have been developed, postoperative weakness of respiratory and limb skeletal muscles has increased, due to stimulation by anesthetic agents, postoperative respiratory tract inflammation and increased release of autoantibodies. This can result in weak cough and expectoration, respiratory weakness, dyspnea, pneumonia, and atelectasis, and can even induce MG crisis [5]. Methods are therefore needed to effectively increase the strength of respiratory muscles, promote cough and expectoration, prevent postoperative pulmonary complications, and improve early postoperative patient quality of life, thereby enhancing early postoperative rehabilitation.

Evidently, there were current studies performing aerobic exercise in patients with myasthenia gravis. It indicated that MG patients can improve their muscular functions by incorporating aerobic and resistance strength training, especially in proximal leg muscles, and physical exercise in myasthenia gravis seemed safe and improved neuromuscular parameters and physical performance-based measures [6, 7]. Meanwhile, physical exercise was also found improved pulmonary function in MG patients, and long-term respiratory muscle endurance training would have better respiratory and functional outcomes [8–10]. It was obvious that aerobic exercise and respiratory muscle training would improve both functional capacity and pulmonary function in MG patients.

More, early postoperative pulmonary rehabilitation training has been reported to promote the recovery of respiratory function in patients with chronic obstructive pulmonary disease and elderly patients with lung cancer [11–13]. Respiratory muscle training after postoperative discharge can also positively affect patients with other pulmonary diseases [14]. Pulmonary rehabilitation training, including moderate- or high-intensity respiratory muscle training (RMT) and aerobic exercise, can have a positive impact on patient outcomes following lung surgery, including reducing postoperative pulmonary complications and duration of hospital stay [15, 16]

Preoperative inspiratory muscle training has also been reported to reduce postoperative pulmonary complications in patients undergoing lung, cardiac and major abdominal surgery [12, 13]. For example, preoperative inspiratory muscle training has been shown to prevent the development of pneumonia and atelectasis in patients undergoing lung resection, coronary artery bypass grafting or heart valve surgery [17–19]. These findings suggested that preoperative rehabilitation interventions, including moderate-to-intense RMT and aerobic exercises, when added to chest physiotherapy, may be an effective management for surgically treated MG patients.

Therefore, the present study evaluated the effects of preoperative moderate-intense RMT and aerobic exercise, in addition to chest physiotherapy, on respiratory vital capacity, exercise capacity, and duration of hospital stay in patients with MG, in order to clarify preoperative respiratory muscle training combined with aerobic exercise improves vital capacity and daily life activity following surgical treatment for MG patients.

## Methods

### Study design and patients

This study was designed as a parallel randomized controlled trial. Eighty patients with MG scheduled for extended thymectomy were randomly divided into study group (SG) and control group (CG). The following inclusion criteria were applied: Patients aged < 65 years with Myasthenia gravis who underwent extended thymectomy (resection of hyperplastic thymus or thymus tumor body) plus mediastinal fat dissection, followed by postoperative insertion of a thoracic drainage tube were enrolled in this study from July 2020 to December 2021 at the First Affiliated Hospital of Sun Yat-sen University. The exclusion criteria of this study were as follows: Patients were excluded if they had significant coronary heart disease, chronic obstructive pulmonary disease, or Joint pain of limbs. The study protocol was approved by the ethics committee of the First Affiliated Hospital of Sun Yat-sen University (No. [2020] 461), and all patients provided written informed consent.

### Preoperative RMT, aerobic exercise and respiratory physiotherapy

Upon admission, each patient was examined by professional rehabilitation therapists and specialized nurses, who evaluated the patient's pulmonary function. Patients randomized to the SG began preoperative moderate aerobic training, including an indoor treadmill and up and down stairs (two sessions per day, 45 min per session, for 5 days), to improve cardiopulmonary function. Patients also started preoperative RMT, including inspiratory muscle training (IMT) with a three-ball breathing trainers (China, SURFTIME). The training was three sessions per day, 15 min per session, for 5 days, and the training workload was up to three balls per inhalation. Meanwhile, chest physiotherapy was started, including practiced coughing and expectorating (three sessions per day, 15 min per session, for 5 days), percussion and vibration of chest wall (three sessions per day, 15 min per session, for 5 days), and forced exhalation (three sessions per day, 15 min per session, for 5 days). Subjects in the CG received only chest physiotherapy preoperatively.

### Postoperative RMT, aerobic exercise and respiratory physiotherapy

Beginning 1 day postoperatively, patients in the SG started daily deep inspiratory training with a respiratory trainer, along with daily abdominal pressure breathing. Patients were placed in the supine position, with sandbags weighing 0.5 kg, 1.0 kg, 2.0 kg, or 5.0 kg or no sandbags placed on their abdomens. Patients were instructed to inhale through their nose and maintain abdominal distension for 2–3 s, followed by retraction of the lips and slow exhale, with an inhalation/exhalation ratio of 1:2–1:3. Muscle strength training of the limbs was performed with American Thera elastic bands (red, medium strength). These exercises consisted of training of the shoulder joints, including forward flexion and abduction and horizontal abduction; training of the hip joints, including forward flexion and abduction; and extension of the knee joints. Initially, each breathing or training exercise was performed 10 times per day, with three times of exercises performed and a 1 min rest time alternately. The numbers of aerobic exercise or respiratory muscle training increased by five per day, with the training lasting for 5 days.

During these training sessions, professional physiotherapists and specialized nurses provided one-to-one guidance to patients, including the proper ways to inhale and exhale and not to hold their breath. Systemic relaxation was performed after training.

Patients in the CG were given a respiratory trainer for deep inhalation training, as well as routine drugs and nursing care as determined by their physicians.

In addition to the above training, patients in both groups received bedside training in sitting up, urination, dressing, directed coughing, and percussion and vibration of chest wall.

### Outcomes

Respiratory parameters were measured before and 7 days after surgery using a pulmonary function equipment (Italy, COSMED Quark PFT3). Parameters measured included vital capacity (VC), forced vital capacity (FEV), forced vital capacity in the first second (FEV1), the ratio of FEV1 to FEV, and peak expiratory flow (PEF).

Exercise capacity was evaluated before and 7 days after surgery using the 6-min walking test (6 MWT). Patients were instructed to walk at their fastest speed on the indicated line without running, and the distance walked at 6 min was recorded [15].

Activities of daily living (ADL) during hospitalization were measured using the modified Barthel index rating scale on days 1, 3 and 5 after surgery. The scores are distributed among 10 items as follows: grooming and bathing (five points each); feeding, toilet use, stair climbing, dressing, bowel management, and bladder management (10 points each); and chair/bed transfer and mobility (15 points each).

### Statistical analysis

Sample size was calculated using the formula for quantitative data pairing: $n = \left[ {\frac{{\left( {z_{{{\alpha \mathord{\left/ {\vphantom {\alpha 2}} \right. \kern-0pt} 2}}} + z_{\beta } } \right)S}}{\delta }} \right]^{2}$. Based on previous experimental results [7], *α* was 0.05, 1–*β* was 0.9, δ was 129.25, and *s* was 128.81. Thus, *n* was calculated to be 13; based on a loss rate of 120%, 40 patients were enrolled in each group. SD were expressed as $\overline{x} \pm s$, wherever applicable. Within-group results were compared by paired *t*-tests, whereas between-group results were compared by independent sample *t*-tests. Sex, however, was compared by chi-square tests. All statistical analyses were performed using SPSS 25.0 statistical software (IBM Company), with *p* ≤ 0.05 regarded as statistically significant [20].

## Results

### Demographic and surgical characteristics

Eighty patients with MG scheduled for surgery were randomly divided into two groups of 40 patients each, a study group (SG) and a control group (CG). None of the patients experienced a postoperative muscle weakness crisis. The demographic and surgical characteristics of the included patients are shown in Table 1. None of these characteristics differed significantly between the SG and CG groups (*p* > 0.05).

**Table 1:** Demographic and surgical characteristics of patients in the SG and CG

Variables	SG	CG	*p*
*n*	40	40	
Age (years)	39.33 ± 12.81	42.94 ± 9.57	0.438
Sex, M/F	19/21	17/23	0.653
Height (cm)	162.33 ± 6.13	160.50 ± 7.27	0.445
Weight (kg)	55.86 ± 6.58	51.50 ± 7.08	0.085
BMI (kg/m^2^)	21.37 ± 2.89	19.98 ± 2.19	0.159
*MGFA classification*			
MGFA I	12 (30.0%)	14 (35.0%)	0.733
MGFA IIA	18 (45.0%)	15 (37.5%)	0.660
MGFA IIB	10 (25.0%)	11 (27.5%)	0.846

*BMI*, body mass index; *CG*, control group; *SG*, studied group

Sex distribution in the two groups was compared using the Pearson chi-square test. All other variables were compared by *t*-tests.表1: 两组间术前比较

### Comparisons of pre- and post-operative respirable vital capacity

Comparisons of pre- and post-operative respirable vital capacity in the two groups are shown in Table 2. Preoperatively, there were no significant differences in parameters of respirable vital capacity between the SG and CG, including in VC, FVC, FEV1, FEV1/FVC, and PEF (*p* > 0.05 each). VC, FVC, FEV1, and PEF decreased significantly after surgical treatment, both in the SG (*p* = 0.001, *p* = 0.001, *p* = 0.002, and *p* = 0.004, respectively) and CG (*p* = 0.001 each). Although pre- and post-operative FEV1/FVC ratios did not differ significantly in either group, these findings demonstrated that surgical treatment would aggravate the weakness of respiratory muscles in patients with MG.

**Table 2:** Comparison of static and dynamic pulmonary function parameters in the SG and CG after surgery

Variables	SG	CG	*p*
*VC (L)*			
Preop	2.87 ± 0.84	3.06 ± 0.74	0.324
Postop	2.09 ± 0.67	1.59 ± 0.36	0.012
*P*	0.001	0.001	
*FVC (L)*			
Preop	2.67 ± 0.90	3.00 ± 0.76	0.345
Postop	1.94 ± 0.60	1.54 ± 0.36	0.030
*P*	0.001	0.001	
*FEV*_*1*_* (L)*			
Preop	2.28 ± 0.86	2.52 ± 0.71	0.395
Postop	1.69 ± 0.57	1.25 ± 0.36	0.014
*P*	0.002	0.001	
*FEV*_*1*_*/FEV (%)*			
Preop	83.39 ± 9.37	87.12 ± 7.35	0.613
Postop	84.85 ± 8.33	82.84 ± 10.16	0.541
*P*	0.141	0.170	
*PEF (L/s)*			
Preop	5.08 ± 1.91	5.52 ± 1.66	0.485
Postop	3.69 ± 0.97	2.84 ± 1.19	0.035
*P*	0.004	0.001	
*6 MWT (m)*			
Preop	554.44 ± 81.17	564.75 ± 109.35	0.764
Postop	499.00 ± 87.62	494.31 ± 122.44	0.902
*P*	0.041	0.001	

*6MWT*, 6-min walking test; *CG*, control group; *FEV1.0*, forced expiratory volume in the first second; *FVC*, forced vital capacity; *PEF*, peak expiratory flow rate; *Preop*, preoperative; *Postop*, postoperative; *SG*, studied group; *VC*, vital capacity

All variables were compared by* t*-tests. *p* ≤ 0.05 was regarded as statistically significant

**Fig. 1:**
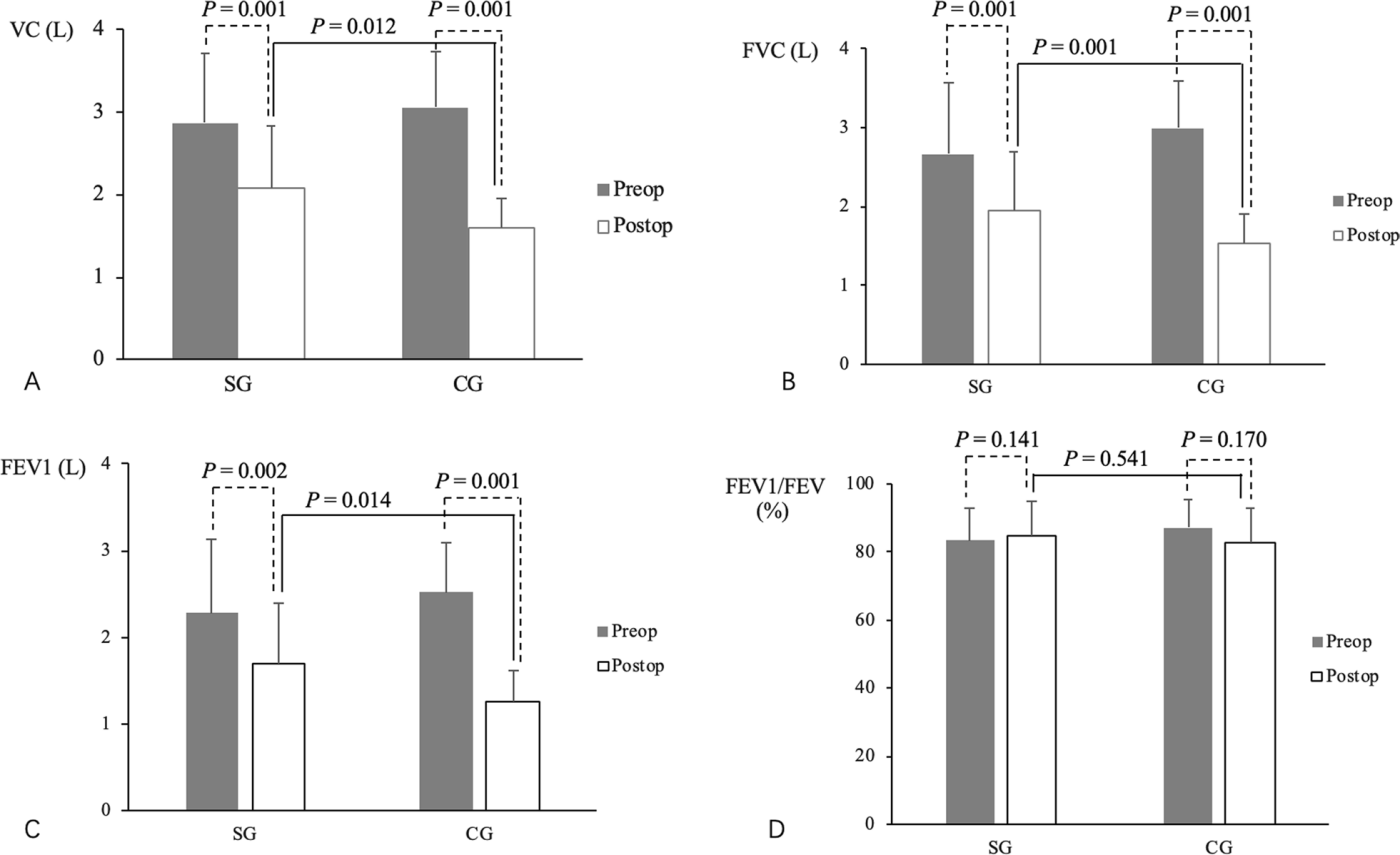
Comparisons of pre- and postoperative indicators of respirable vital capacity in the SG and CG. **A** VC; **B** FVC; **C** FEV1; **D** FEV1/FVC. Abbreviations: CG, control group; FEV1.0, forced expiratory volume in the first second; FVC, forced vital capacity; Preop, preoperative; Postop, postoperative; SG, studied group; VC, vital capacity

Pre- and postoperative RMT and aerobic exercise were found to have a beneficial effect on parameters of respirable capacity (Fig. 1 and Table 2). Comparisons showed that VC (2.09 ± 0.67 l vs 1.59 ± 0.36 L, *p* = 0.012), FVC (1.94 ± 0.60 l vs 1.54 ± 0.36 L, *p* = 0.030), FEV1 (1.69 ± 0.57 l vs 1.25 ± 0.36 L, *p* = 0.014), and PEF (3.69 ± 0.97 L/S vs 2.84 ± 1.19 L/s, *p* = 0.035) were significantly higher in the SG than in the CG, although their FEV1/FVC ratios did not differ significantly (*p* > 0.05).

### Comparisons of pre- and post-operative exercise capacity

Comparisons of pre- and post-operative exercise capacity are shown in Table 2. Preoperatively, results on the 6MWT did not differ significantly in the SG and CG (*p* > 0.05). Following surgical treatment, however, times on the 6 MWT decreased significantly in both the SG (*p* = 0.041) and CG after surgical treatment (*p* = 0.001), indicating that surgical treatment exacerbated the weakness of limb skeletal muscles in patients with MG.

Postoperatively, results on the 6MWT did not differ significantly between the SG and CG (*p* > 0.05) (Fig. 1, Table 2). These findings indicated that pre- and postoperative RMT and aerobic exercise did not affect the surgery-associated reduction in limb skeletal muscle and exercise capacity in patients with MG.

### Comparisons of pre- and post-operative ADL during hospitalization

**Fig. 2:**
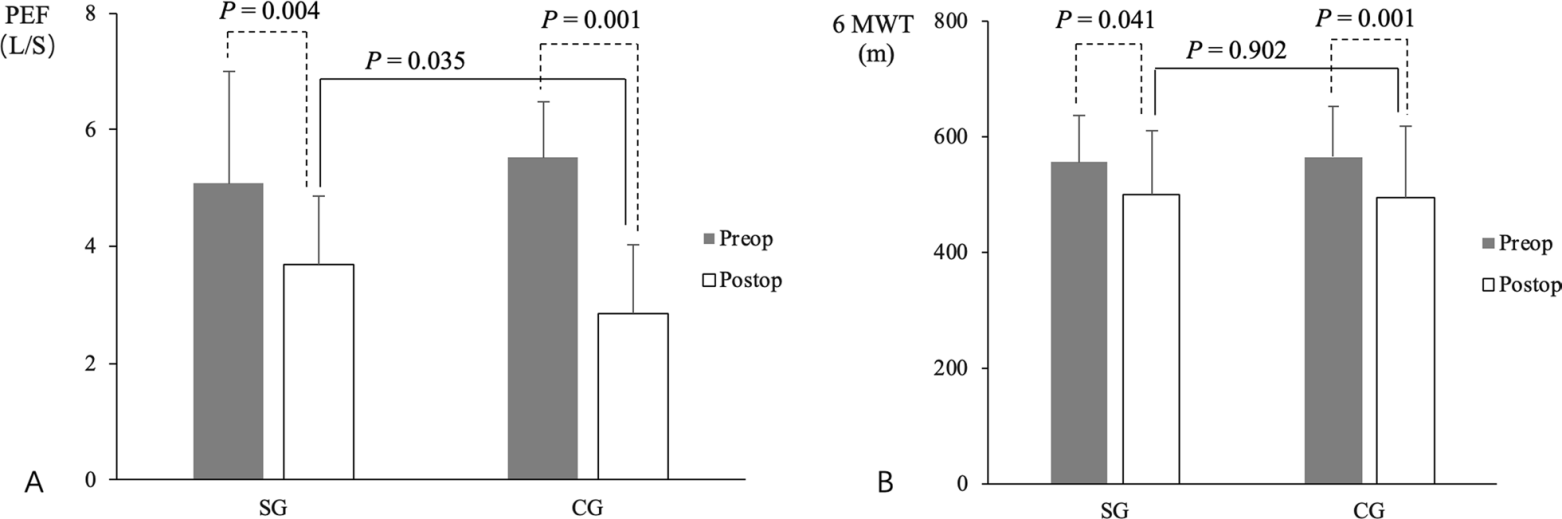
Comparisons of pre- and postoperative **A** PEF and **B** exercise capacity, as determined by the 6MWT, in the SG and CG. Abbreviations: 6MWT, 6-min walking test; CG, control group; PEF, peak expiratory flow rate; Preop, preoperative; Postop, postoperative; SG, studied group

**Fig. 3:**
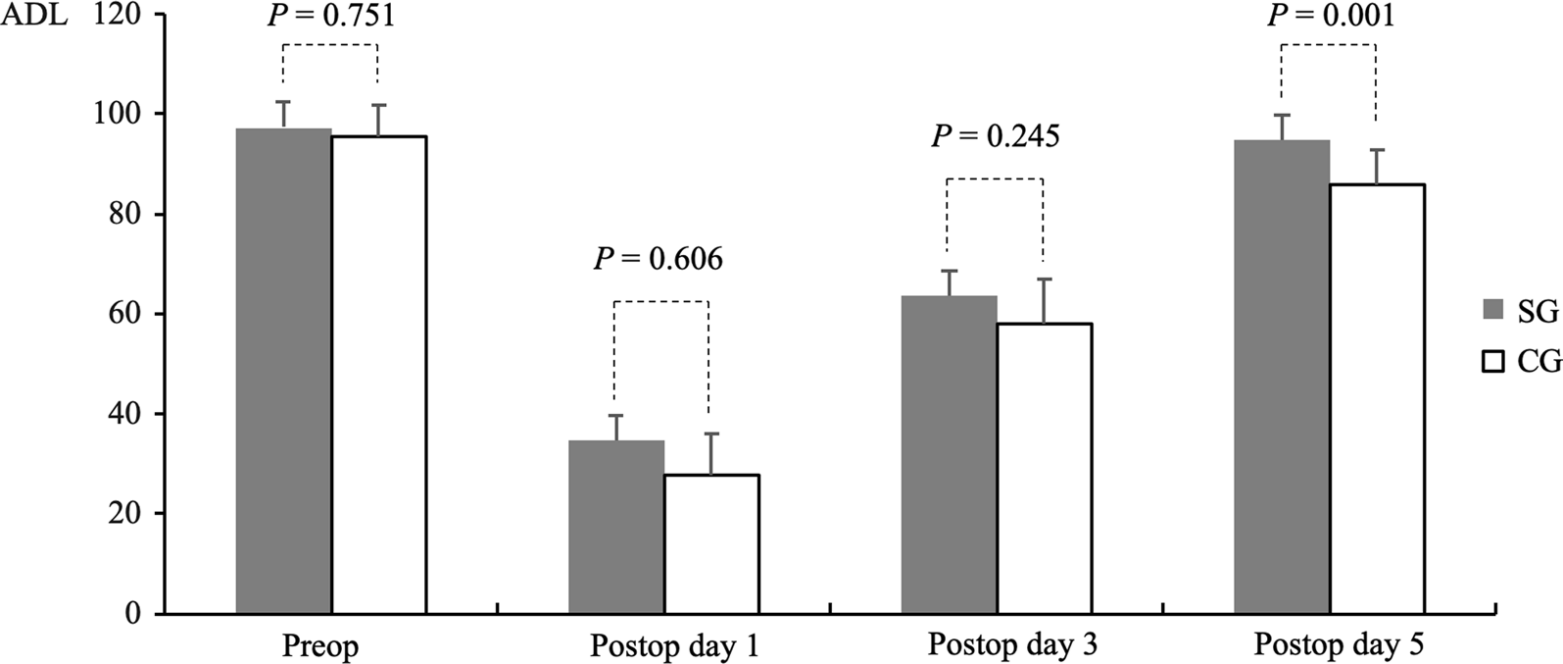
Comparisons of pre- and postoperative ADL during hospitalization in the SG and CG. Abbreviations: ADL, activities of daily life; CG, control group; Preop, preoperative; Postop, postoperative; SG, studied group

Comparisons of pre- and post-operative ADL are shown in Table 3 and Fig. 2. Preoperatively, ADL did not differ significantly between the SG and CG (97.32 ± 5.19 vs 97.51 ± 6.40, *p* > 0.05). ADL was markedly lower in both the SG and CG 1 day (34.76 ± 15.69 vs 27.92 ± 8.11, *p* > 0.05) and 3 days (63.57 ± 13.89 vs 57.92 ± 8.91, *p* > 0.05) after surgical treatment, although the differences between these groups were not statistically significant. These findings indicated that surgical treatment had a negative effect on ADL in patients with MG. Five days after surgical treatment, however, ADL increased to preoperative levels in the SG and was significantly higher than in the CG (94.76 ± 4.87 vs 85.83 ± 7.02, *p* = 0.001). These findings indicated that pre- and postoperative RMT and aerobic exercise had a positive influence on the postoperative recovery of ADL in patients with MG (Fig. 3).

**Table 3:** Comparison of ADL in the SG and CG during hospitalization

Variables	SG	CG	*p*
Preoperative ADL	97.32 ± 5.19	97.51 ± 6.40	0.751
*Postoperative ADL*			
Day 1	34.76 ± 5.69	27.92 ± 8.11	0.606
Day 3	63.57 ± 3.89	57.92 ± 8.91	0.245
Day 5	94.76 ± 4.87	85.83 ± 7.02	0.001

*ADL*, activities of daily life; *CG*, control group; *SG*, studied group

All variables were compared by* t*-tests. *p* ≤ 0.05 was regarded as statistically significant

## Discussion

MG is a rare neuromuscular disorder characterized by muscle weakness and fatigue that is related to pathology of the thymus. The muscle weakness of MG not only involves the eyelid muscles, but can also involve the respiratory, limb skeletal, and swallowing muscles. MG is exacerbated by anesthetic drugs, postoperative respiratory tract inflammation, emotional alterations, and increased autoantibody release, resulting in weak cough and expectoration, respiratory weakness, dyspnea, pneumonia, and atelectasis, and can even induce MG crisis [5, 21, 22]. Although there were ten intervention studies of the safety and usefulness of systematic training in MG patients, and three of them on respiratory muscles [23], it is unclear whether active training of respiratory muscle groups can improve their postoperative strength and reduce postoperative respiratory complications in patients with MG. The present study therefore evaluated the effects of preoperative moderate-to-intense RMT and aerobic exercise, when added to chest physiotherapy, on respiratory vital capacity, exercise capacity, and duration of hospital stay in patients with MG, and showed that RMT and aerobic exercise can have positive effects on postoperative respiratory vital capacity and daily life activity, and would enhance recovery after surgery in MG patients.

Perioperative accelerated rehabilitation has been shown safe in patients following thoracic surgery, reducing postoperative complications and hospital stay. American Association of Chest Physicians (ACCP) guidelines for rehabilitation diagnosis and treatment of lung diseases recommend the optimization of perioperative treatment. Methods recommended to reduce trauma stress and complications and shorten hospital stay include the use of multi-modal methods to accelerate patient rehabilitation, such as getting out of bed soon after surgery, shortening the duration of fasting before the operation and oral feeding soon after surgery [24, 25]. Because surgical treatment for MG uses similar anesthetic drugs and surgical intervention environments as lung resection, and because thymectomy has become minimally invasive, the degree and duration of postoperative pain have been significantly reduced and patients can get out of bed significantly earlier. MG patients undergoing surgery may therefore benefit from preoperative moderate-to-intense RMT and aerobic exercises, in addition to chest physiotherapy.

More importantly, previous study showed exercise and repetitive muscle use have been reported to aggravate muscle weakness in patients with MG, suggesting that exercise may be harmful for such patients [26]. In contrast, the present study showed that moderate-to-intense RMT plus chest physiotherapy can have positive effects on postoperative respiratory vital capacity and ADLs in surgically treated MG patients. Previous studies have tested several rehabilitative approaches, including physical, respiratory, and balance training, with results suggesting that a multidisciplinary rehabilitation approach would be better than any single approach in patients with MG, especially those with mild to moderate symptomatology [6, 7, 9, 27–34]. The benefits of respiratory training were shown to include not only measurable improvements in respiratory muscle strength, respiratory endurance, and physical performance [6, 7, 9, 27–34]. but also reductions in the incidence of several complications of MG, including dyspnea [35]. Because it was found to significantly reduce fatigue in the diaphragm and skeletal muscles, sustained hyperpnea training has been considered more appropriate than respiratory strength training in patients with MG [7, 32, 35].

The present study also showed that pre- and postoperative RMT and aerobic exercise had no effects on the decreases in limb skeletal muscle and exercise capacity caused by surgery in MG patients. The absence of effects may be associated with the relatively short duration of RMT and weaknesses in limb skeletal muscles specifically associated with MG. Although the duration of hospital stay in patients undergoing lung surgery following preoperative exercise training ranged from 4 days to 2 weeks [13] and patients receiving preoperative exercise before surgery reported an improved exercise capacity with an increase in distance on the 6MWT [33], the present study provided no evidence that preoperative exercise enhanced recovery of exercise capacity after surgery in patients with MG.

Most importantly, although the previous studies indicated that aerobic exercise and respiratory muscle training would improve both functional capacity and pulmonary function in MG patients, the present study also found that the ADL 5 days after surgery was significantly better in the SG than in the CG, although the differences 1 and 3 days after surgery were not statistically significant. Similarly, physical activity after thoracic surgery was reported to be very limited 1–4 days after surgery, with pain and dizziness being the main factors affecting early postoperative movement [36–39]. Subjects with reduced pain postoperatively were encouraged to get out of bed and start rehabilitation training as soon as possible, resulting in improved ADL.

On the other hand, the present study was a small sample randomized parallel controlled trail. Regarding the small sample size, it is necessary to increase the sample size to verify the positive effects of RMT and aerobic exercise on postoperative respiratory vital capacity and daily life activity in MG patients. Additional studies are required to test the effectiveness and safety of rehabilitation in surgically treated MG patients.

In summary, the present study showed that preoperative moderate-to-intense RMT and aerobic exercise, in addition to chest physiotherapy, can have positive effects on postoperative respiratory vital capacity and daily life activities in patients with MG. No side effects have been linked to physical training in individuals. These findings indicate the need to accurately predict improvements in exercise tolerance in surgically treated MG patients.
